# Automated L3 Skeletal Muscle Segmentation for the Evaluation of Sarcopenia: Development and Independent Validation of an Ensemble-Based 2D nnU-Net Pipeline in a Complex Liver Disease Cohort

**DOI:** 10.3390/muscles5020040

**Published:** 2026-06-03

**Authors:** Hyeon Yu, Kevin Wang

**Affiliations:** Division of Vascular and Interventional Radiology, Department of Radiology, University of North Carolina at Chapel Hill, 2018 Old Clinic, CB 7510, Chapel Hill, NC 27599, USA

**Keywords:** sarcopenia, skeletal muscle segmentation, computed tomography, deep learning, liver cirrhosis

## Abstract

Purpose: To develop a fully automated 2D nnU-Net pipeline for multi-class skeletal muscle segmentation (psoas, paraspinal, and abdominal wall) at the third lumbar (L3) vertebral level, and to quantitatively evaluate its diagnostic performance and reliability compared to manual segmentation. Materials and Methods: A 2D nnU-Net was trained on 164 axial L3 CT slices from the multi-institutional AMOS22 dataset, spanning diverse abdominal pathologies and multivendor imaging. To assess generalizability under severe anatomical distortion, independent external validation was performed in 50 consecutive patients with advanced liver disease from a single institution (January–December 2025; mean age, 63 ± 15 years; 32 women, 18 men), of whom 88% had moderate-to-severe ascites. Model stability was examined by comparing a five-fold ensemble with the best-performing single-fold model. Intra-observer reliability of the manual reference standard was evaluated in a random subset of 30 cases. Inter-observer agreement was additionally assessed using an independent second reader. Performance metrics included the Dice Similarity Coefficient (DSC), Pearson correlation coefficient (r), and Bland–Altman analysis for cross-sectional areas and mean attenuation. The inference workflow was deployed via a custom Streamlit-based graphical user interface (GUI). Results: In this anatomically complex external validation cohort, the 5-fold ensemble 2D nnU-Net achieved an overall mean DSC of 0.937 ± 0.043 (95% CI, 0.925–0.950), with 80% of cases achieving a mean DSC ≥ 0.90. While the mean DSC was statistically comparable to the best single-fold model (0.937, [95% CI, 0.921–0.952], *p* = 0.736), the ensemble strategy increased the minimum observed DSC (worst-case performance) from 0.720 to 0.822. Class-specific external validation performance for the 5-fold ensemble was highest for the paraspinal muscles (DSC: 0.960; 95% CI, 0.952–0.967), followed by the psoas muscles (DSC: 0.941; 95% CI, 0.927–0.956), and lowest for the anatomically complex abdominal wall muscles (DSC: 0.911; 95% CI, 0.893–0.929). Comparison between the ensemble model and manual segmentation yielded a Pearson correlation of r = 0.955 (*p* < 0.001) for total skeletal muscle area, with a mean bias of +7.17 cm^2^. Intra- and inter-observer agreements for the manual reference standard demonstrated correlation coefficients of r = 0.995 and 0.090 for total areas, respectively. The automated pipeline required 3–5 s per case for inference and quantitative reporting, compared to 3–5 min for manual segmentation. Conclusions: In patients with advanced liver disease and substantial anatomical distortion from ascites, an ensemble-based 2D nnU-Net provides high quantitative agreement with manual L3 skeletal muscle segmentation, while mitigating lower-bound (worst-case) errors relative to single-fold models. Integration with a dedicated GUI enables substantial time savings and supports scalable quantitative body composition measurement.

## 1. Introduction

Sarcopenia, a term initially proposed by Irwin H. Rosenberg to describe age-related muscle loss, has become a critical focal point across diverse medical disciplines [1]. The European Working Group on Sarcopenia in Older People (EWGSOP) formally defines sarcopenia as a syndrome characterized by the generalized and progressive loss of skeletal muscle mass and strength, which is associated with adverse outcomes, including reduced quality of life and increased mortality [2,3,4,5,6,7,8]. While sarcopenia is estimated to affect 5 to 10% of the population over the age of 65 years in the context of primary geriatric frailty, opportunistic body composition analysis has demonstrated its broader clinical significance [2,9,10,11,12,13,14]. Reduced skeletal muscle mass is now recognized as an independent predictor of adverse clinical outcomes across diverse patient populations, leading to increased rates of surgical complications, higher chemotherapy toxicity, prolonged hospital length of stay, and decreased overall survival in patients with malignancies, end-stage liver disease, and major vascular conditions [15,16,17,18,19,20,21,22,23].

The quantitative assessment of sarcopenia is most commonly performed using cross-sectional imaging, specifically computed tomography (CT) [16,17,24,25,26,27,28,29]. The skeletal muscle cross-sectional area (SMA) measured at the level of the third lumbar vertebra (L3) serves as the established reference standard, as it exhibits a high linear correlation with total body skeletal muscle volume [3,12,26,30,31,32,33]. At this anatomical landmark, the target musculature comprises three distinct groups: the psoas, the paraspinal (erector spinae and quadratus lumborum), and the abdominal wall muscles (transversus abdominis, internal and external obliques, and rectus abdominis) [26,31,33,34]. Despite the proven prognostic value of L3 skeletal muscle quantification, its integration into routine clinical practice remains limited by methodological bottlenecks [4,5,35]. The current reference standard for extracting these metrics relies on manual or semi-automated segmentation using third-party analytical software (e.g., Slice-O-Matic, ImageJ, or Horos) [36,37,38]. These conventional tools primarily utilize Hounsfield Unit (HU) thresholding—typically applying a predefined window of −29 to +150 HU—to isolate skeletal muscle from surrounding adipose tissue and viscera [4,27].

While HU thresholding is effective in healthy anatomies, it frequently fails in complex clinical scenarios [39,40]. In patients with sarcopenia, the abdominal wall muscles often become severely thinned and undergo myosteatosis (fatty infiltration), which alters their radiodensity [26,33,34,41]. Furthermore, the presence of abdominal wall edema, ascites, or closely apposed bowel loops creates anatomical interfaces with attenuation values that overlap those of skeletal muscle [39,42,43]. In these instances, thresholding algorithms cannot accurately delineate the peritoneal boundary, necessitating extensive manual correction by a human reader [39]. This manual tracing process is highly labor-intensive, often requiring 3 to 5 min per axial slice, thereby introducing inter-observer variability and precluding large-scale, population-level opportunistic screening [44,45,46]. To address the limitations of manual segmentation, deep learning (DL) algorithms, particularly convolutional neural networks (CNN) based on the U-Net architecture, have been increasingly applied to medical image segmentation [35,44,45,46,47,48]. The nnU-Net framework, a self-configuring architecture, currently represents the state of the art for such tasks, automating hyperparameter tuning and preprocessing to achieve high spatial accuracy [49,50,51,52,53].

However, translating these high-performing theoretical models into reliable clinical tools presents ongoing challenges [54,55,56]. A review of current literature indicates that many published DL models for body composition are trained and validated on relatively homogenous, single-center datasets or heavily index toward healthy outpatient populations [39,45,47,57,58]. When deployed on external datasets containing high-acuity pathologies—such as massive tumors, postoperative anatomical distortion, or severe fluid shifts—these models are susceptible to severe segmentation failures [40,45,59,60]. In a clinical quality and safety context, these unpredictable lower-bound errors limit the trustworthiness of fully automated pipelines [60,61]. Most existing DL approaches utilize a single-fold model architecture, which lacks a consensus mechanism to resolve anatomical ambiguity at the muscle-peritoneum interface [49,62,63].

To bridge the gap between technical DL performance and safe clinical deployment, robust segmentation pipelines must be validated on complex, multi-vendor clinical data. Furthermore, to achieve efficient workflow integration, these technical pipelines must be accessible through intuitive software interfaces. Therefore, this study was initiated to develop and independently validate a fully automated 2D nnU-Net pipeline for L3 skeletal muscle segmentation. To maximize model generalizability, the training phase utilized a heterogeneous, multi-institutional dataset containing diverse abdominal pathologies. Additionally, to rigorously test algorithmic stability under severe anatomical distortions, external validation was conducted on a challenging clinical cohort of patients with advanced liver disease. To address unpredictable segmentation failures, we implemented a 5-fold ensemble inference strategy to stabilize boundary predictions. Finally, we integrated this ensemble model into a custom graphical user interface (GUI) to evaluate its impact on measurement reliability and workflow efficiency compared to the conventional manual reference standard.

## 2. Materials and Methods

### 2.1. Study Design and Patient Cohorts

This retrospective study used two distinct datasets for model development and independent external validation. For model training and internal cross-validation, 164 axial CT slices at the L3 vertebral level were extracted from the AMOS22 (Abdominal Multi-Organ Segmentation) dataset, a multi-institutional, multivendor resource. Metadata analysis of the training pool showed a diverse distribution of scanner manufacturers (Toshiba, Siemens, GE Healthcare, and Philips Healthcare) and a mean patient age of 53.7 ± 16.3 years. To promote robustness to anatomical distortion, we purposefully selected cases representing a heterogeneous spectrum of abdominal pathologies, including ascites, primary and metastatic hepatic malignancies, renal tumors, and peritoneal carcinomatosis. Only axial L3 slices with interpretable anatomy and adequate visualization of the target muscle compartments were retained for manual annotation and model development. The AMOS22-derived training cohort included CT examinations acquired across multiple scanner manufacturers and institutions, thereby introducing heterogeneity in acquisition conditions during model development. However, the present study did not perform predefined subgroup analysis stratified by scanner vendor, reconstruction parameters, contrast phase, or acquisition protocol.

The independent external validation cohort (*n* = 50) was retrospectively identified from an existing Institutional Review Board–approved study evaluating predictors of transjugular intrahepatic portosystemic shunt (TIPS) revision in patients with advanced liver disease (Table 1). As a result, clinical indications for imaging were clustered around chronic hepatic pathology; the most common were liver cirrhosis (22%), routine TIPS evaluation (20%), and refractory ascites (18%). From an algorithmic perspective, this cohort was designed to stress-test the segmentation pipeline: patients with end-stage liver disease frequently exhibit compounding anatomical distortions—severe macroscopic sarcopenia, massive ascites, subcutaneous abdominal edema, and altered fascial planes—that pose challenging edge cases for both manual tracing and automated boundary delineation.

Exclusion criteria for the external validation cohort were:

- CT examinations in which the L3 axial slice did not encompass complete abdominal coverage,
- DICOM studies that could not be exported due to PACS technical issues,
- Absence of abdominal CT (abdominal MRI only), and
- Ineligibility related to the source database (patients who did not undergo a TIPS procedure). No attempt was made to impute missing anatomy or reconstruct incomplete slices; studies not meeting these eligibility criteria were excluded before annotation and model evaluation.

### 2.2. Image Preprocessing and Reference Standard Generation

To mitigate inter-reader variability and standardize the segmentation workflow, a custom GUI was developed using the Streamlit framework (version 1.30.0, https://streamlit.io/) in Python (version 3.10). All L3 axial CT images were loaded into the GUI and preprocessed using a standard soft-tissue window (window width: 400 HU; window level: 50 HU) to optimize the visualization of muscle-adipose interfaces. A board-certified radiologist (HY) generated manual reference standards utilizing a custom brush-based annotation interface within the Streamlit application. Three distinct target classes were sequentially delineated and consolidated into a unified label map: the psoas muscle (class 1), the paraspinal muscles (class 2), and the abdominal wall muscles (class 3), with the background designated as class 0. Slices with an incomplete field-of-view at the L3 level were not annotated. Likewise, missing regions were not estimated by interpolation or extrapolation; only directly visible anatomy was used to generate the reference standard.

Upon completion of each case, the application automatically exported and archived the original CT slice and the corresponding final label map as a pair of Neuroimaging Informatics Technology Initiative (NIfTI) files (*-image.nii.gz and *-label.nii.gz). This standardized nomenclature and label identification protocol ensured direct compatibility for subsequent dataset curation and model training. Concurrent with mask generation, case-level quantitative logs—including total and class-specific cross-sectional area (cm^2^), mean attenuation (HU), and attenuation standard deviation—were extracted to serve as the reference standard.

To establish the stability of this manual reference standard and mitigate recall bias, intra-observer reliability was quantitatively assessed. The same board-certified radiologist performed a second, independent manual segmentation round on a random subset of 30 cases from the initial AMOS22 training dataset. This second annotation session was conducted after a minimum 14-day washout period, and the rater was strictly blinded to the initial segmentation results and clinical metadata. To further assess inter-observer variability, an independent second reader manually segmented a random subset of 30 cases from the initial AMOS22 training dataset using the same Streamlit-based workflow and class definitions. Agreement between the two readers was evaluated using class-specific Dice coefficients and quantitative agreement metrics for cross-sectional areas and mean attenuation.

### 2.3. Model Architecture and Training Protocol

An automated segmentation model was developed using the 2D configuration of the nnU-Net v2 framework. The nnU-Net utilizes a self-configuring U-Net encoder–decoder architecture that automatically dynamically adapts image preprocessing, network topology, and training hyperparameters based on the heuristic properties of the training dataset.

Model training was executed on a local workstation equipped with an NVIDIA GeForce RTX 4090 graphics processing unit (24 GB VRAM). Training was conducted using a 5-fold cross-validation strategy. To implement this, the 164-case training dataset was partitioned into five non-overlapping subsets (folds). The network was trained five times; in each iteration, four folds (80% of the data) were used for active training, while the remaining fold (20%) was held out for internal validation. Model training used the standard nnU-Net v2 on-the-fly augmentation pipeline for 2D semantic segmentation. During training, augmented samples were generated dynamically rather than stored as precomputed images. The augmentation framework included randomized spatial transformations and image-intensity perturbations automatically configured by nnU-Net for the dataset and network configuration, thereby exposing the model to plausible variations in muscle shape, boundary position, and soft-tissue contrast during each epoch. This dynamic augmentation strategy was used consistently across all cross-validation folds.

This rotational sampling ensures that every image in the dataset is used exactly once as unseen validation data, thereby mitigating the risk of overfitting and providing a robust, unbiased estimate of internal segmentation accuracy. Each fold was trained for 1000 epochs, requiring an average computational time of 7.3 to 7.7 h per fold (average epoch duration of approximately 26 s).

The network was optimized using a batch size of 6, an image patch size of 640 × 640 pixels, and a resampled in-plane voxel spacing of 0.782 × 0.782 mm. Image intensities were standardized utilizing the framework’s dedicated CT normalization scheme (CTNormalization), which automatically clips Hounsfield Unit (HU) values based on the global foreground statistics of the training cohort. Standardized data augmentation techniques, including random rotations, scaling, and Gaussian noise addition, were applied during training to enhance the model’s resilience to variations in scanner protocols and image reconstruction algorithms.

Following the cross-validation phase, an ensemble inference strategy was employed to process the external validation cohort. Instead of relying on a single network hierarchy, the output probability maps generated by all five independently trained folds were mathematically averaged. This approach subsequently yielded a final, stable label map for each unseen clinical case, thereby mitigating the risk of localized boundary failures associated with single-model predictions.

### 2.4. Automated Inference Workflow and User Interface

To facilitate clinical evaluation and streamline future dataset curation, the trained 2D nnU-Net models were deployed within the local Streamlit application. This end-to-end clinical workflow consists of four primary functions: (1) direct importation of unannotated single-slice CT images; (2) manual multi-class segmentation with brush-based editing capabilities; (3) on-demand execution of automated 2D nnU-Net inference, allowing the user to select either the best-performing single-fold network or the 5-fold ensemble architecture to generate initial predictive or comparison masks; and (4) real-time review of quantitative outputs. During model testing, the application automatically computes spatial overlap metrics when a reference mask is present, alongside immediate area and attenuation summaries. For each completed case, the application exports the original image and final label map as a paired NIfTI set (*-image.nii.gz, *-label.nii.gz) with consistent naming and label IDs, enabling direct reuse of outputs for dataset curation and iterative model testing.

### 2.5. Statistical Analysis

Quantitative evaluation of spatial overlap between the automated segmentations and the manual reference standard was performed using the Dice Score and Dice Similarity Coefficient (DSC). The Dice Score between the predicted mask P and the reference mask G was defined as shown in Equation (1):

(1) $$\mathrm{DSC}\left(P,G\right)=\frac{2(P\cap G)}{\left|P|+|G\right|}$$

where P represents the predicted segmentation, G represents the manual ground truth, ∣P∩G∣ denotes the number of voxels shared by both masks, and ∣P∣ and ∣G∣ correspond to the number of voxels labeled positive in the predicted and manual masks, respectively.

The equivalent formulation in terms of classification performance was also computed, as shown in Equation (2):

(2) $$\mathrm{DSC}=\frac{2TP}{2TP+FP+FN}$$

where TP, FP, and FN indicate the number of true-positive, false-positive, and false-negative voxels, respectively. Performance was analyzed for each individual muscle class as well as the overall mean across all foreground classes. For the external validation cohort, 95% confidence intervals for the mean DSC values were estimated from the case-level DSC distributions using the t-distribution.

Clinical utility was assessed by extracting the total cross-sectional area (cm^2^) and mean attenuation (HU) from the generated label maps. The agreement between the 2D nnU-Net measurements and the manual reference measurements was evaluated using the Pearson correlation coefficient (r) and Mean Absolute Error (MAE). Bland–Altman analysis was conducted to determine the mean bias and the 95% limits of agreement between the two methods.

To quantify the benefit of the ensemble architecture, a paired *t*-test was used to compare the mean DSC of the 5-fold ensemble model with that of the single best-performing fold from the cross-validation phase. Statistical significance was defined as a *p*-value < 0.05. This inferential comparison was performed at the case level within the independent external validation cohort. Internal five-fold cross-validation metrics were treated as descriptive estimates of training stability and reproducibility across partitions rather than as statistically independent groups for formal hypothesis testing.

Quantitative metrics were extracted from NIfTI label maps using custom Python scripts, employing the nibabel library (version 5.3.2) for medical image input/output and numpy (version 2.4.2) for numerical array operations. All subsequent tabular data aggregation and statistical analyses were conducted utilizing pandas (version 2.2.3) and scipy.stats module within the scipy library (version 1.17.0). Graphical visualizations, including box plots, correlation plots, and Bland–Altman plots, were generated using the matplotlib (version 3.9.1) and seaborn (version 0.13.2) libraries.

## 3. Results

### 3.1. Internal Cross-Validation Performance

**Figure 1:**
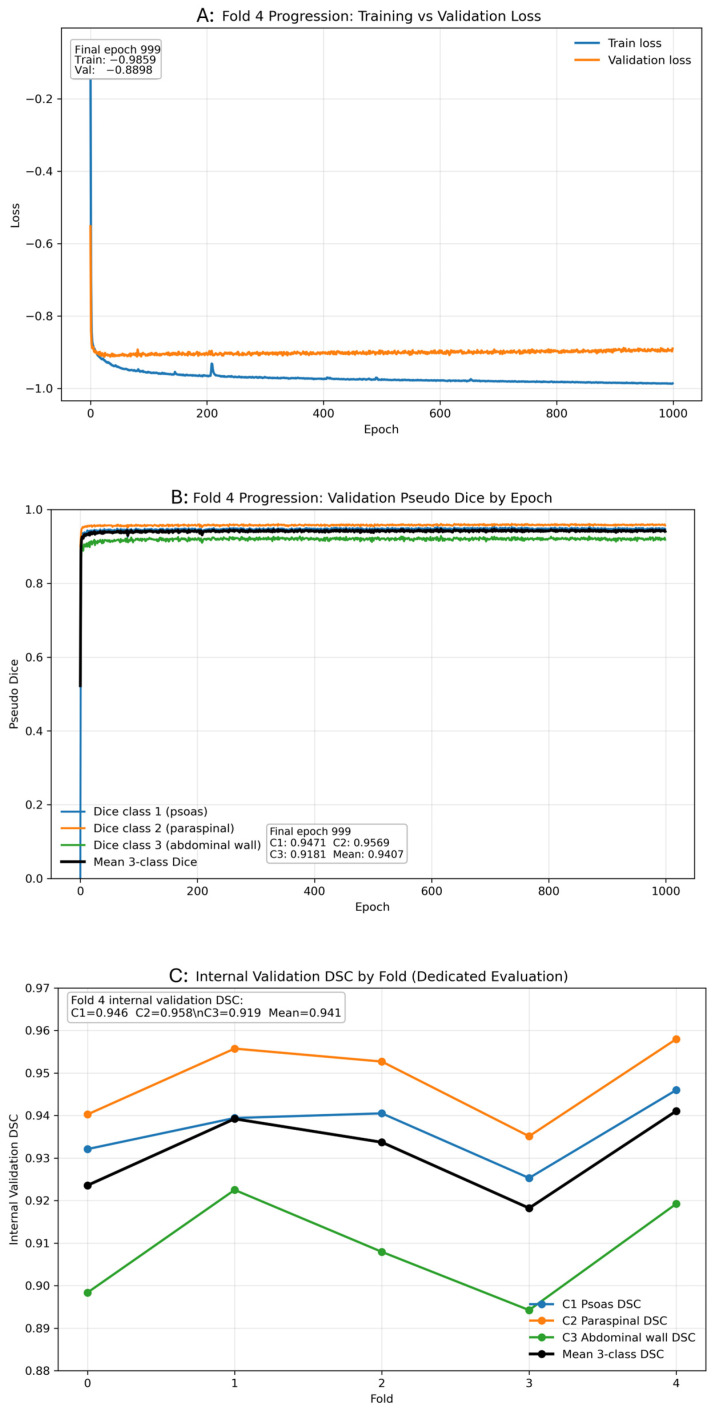
Training convergence and internal cross-validation performance of the 2D nnU-Net. (**A**) Training and validation loss progression over 1000 epochs for a representative cross-validation partition (Fold 4). Both loss curves exhibit steady minimization and parallel plateauing, indicating optimal convergence and the absence of model overfitting. (**B**) Progression of the validation pseudo-Dice Similarity Coefficient (DSC) evaluated at the end of each epoch for the same representative fold. The curves detail the independent stabilization of the individual target classes—psoas (class 1), paraspinal (class 2), and abdominal wall (class 3)—alongside the overall 3-class mean. (**C**) Final internal validation DSC evaluated across all five independent partitions (Folds 0–4) from the cross-validation phase. The line plot illustrates the performance consistency of the network architecture across different subsets of the dataset. Spatial overlap for individual anatomical classes and the overall mean remains stable across all folds, with Fold 4 achieving the highest internal validation performance prior to external testing.

During the initial development phase, the 2D nnU-Net model demonstrated high internal consistency across the 164-case training cohort. In the 5-fold cross-validation, the overall mean foreground Dice similarity coefficient (DSC) was 0.931 ± 0.010 (Table 2). Spatial overlap was highest for the paraspinal muscle group (DSC: 0.948 ± 0.010), followed by the psoas muscles (DSC: 0.937 ± 0.008), and the anatomically complex abdominal wall muscles (DSC: 0.908 ± 0.012). These fold-wise results were used to assess performance stability across training partitions and to identify the highest-performing single fold for subsequent comparison with the ensemble model, rather than as independent samples for formal between-fold hypothesis testing. This internal class-specific ordering was preserved during external validation, indicating that the abdominal wall remained the most challenging target compartment despite overall strong model performance. In addition, the training and validation loss curves showed parallel convergence without late divergence in the representative fold analysis, suggesting stable optimization without severe overfitting during training. These internal validation metrics established a robust baseline for subsequent external testing (Figure 1).

### 3.2. External Validation and Ensemble Efficacy

**Figure 2:**
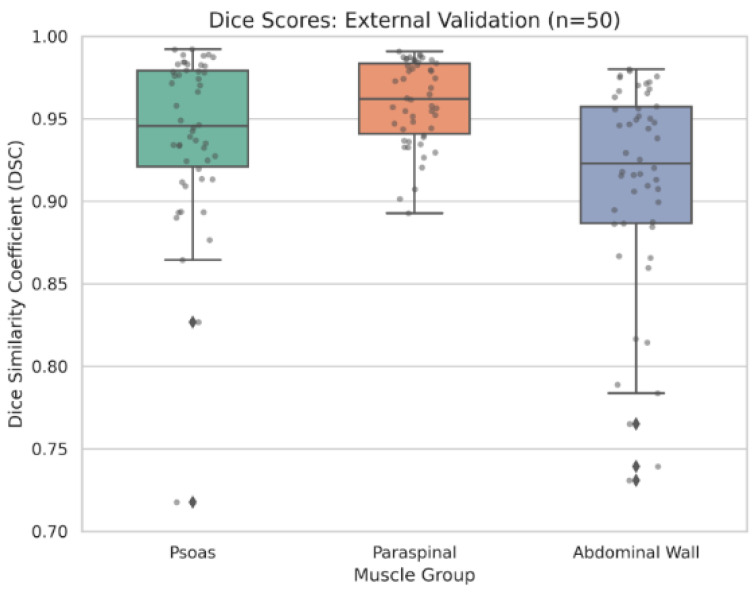
The box-and-whisker plot illustrates the segmentation accuracy of the 5-fold ensemble 2D nnU-Net across 50 consecutive clinical cases. Horizontal lines denote the median Dice Similarity Coefficient (DSC), boxes represent the interquartile range (IQR), and whiskers extend to 1.5 times the IQR. Gray circles represent individual cases, and diamond symbols indicate outliers beyond the whiskers. The high median performance across all three muscle classes establishes the model’s generalizability to high-acuity, multi-vendor data.

Independent external validation was conducted on the 50-case clinical cohort. The 5-fold ensemble 2D nnU-Net model achieved an overall mean DSC of 0.937 ± 0.043 (95% CI, 0.925–0.950) across all muscle classes, demonstrating high generalizability to multi-vendor, unseen data (Figure 2). Performance remained stratified by anatomical complexity: paraspinal DSC was 0.960 (95% CI, 0.952–0.967), psoas DSC was 0.941 (95% CI, 0.927–0.956, and abdominal wall DSC was 0.911 (95% CI, 0.893–0.929) (Table 3). This class-specific performance gradient indicates that the network was most robust in the paraspinal compartment, intermediate in the psoas muscles, and least robust in the abdominal wall, where severe muscle thinning, edema, and poorly defined peritoneal interfaces frequently increased boundary ambiguity.

To evaluate the utility of the ensemble architecture, performance was compared against the single best-performing fold from the internal validation phase (Fold 4). While the overall mean DSC between the best single fold and the 5-fold ensemble was statistically comparable (0.937 vs. 0.937, *p* = 0.736), the ensemble strategy demonstrated a measurable impact on segmentation stability in challenging cases (Table 3). Specifically, the ensemble approach increased the minimum observed DSC within the cohort—representing the “worst-case” segmentation—from 0.720 (single fold) to 0.822 (ensemble). Accordingly, formal statistical model comparison was based on paired case-level performance in the external validation cohort, where each case was evaluated by both inference strategies under identical reference conditions. This finding is clinically relevant because lower-bound failures, rather than average cohort performance, are more likely to determine safety and trustworthiness during real-world implementation.

To further characterize the clinical reliability of the automated pipeline, the proportion of cases achieving a high-performance threshold (DSC ≥ 0.90) was evaluated. For the 5-fold ensemble model, 80% (40/50) of cases achieved an overall mean DSC ≥ 0.90 (sub-threshold mean: 0.868, n = 10). At the class level, performance was most robust in the paraspinal musculature, where 98% (49/50) of cases exceeded the threshold; the single sub-threshold case maintained a high baseline DSC of 0.893. The psoas group achieved a DSC ≥ 0.90 in 84% (42/50) of cases (sub-threshold mean: 0.857, n = 8). The abdominal wall presented the greatest anatomical challenge, exceeding the 0.90 threshold in 68% (34/50) of cases, though the 16 sub-threshold cases still maintained a clinically acceptable mean DSC of 0.836.

### 3.3. Clinical Agreement and Bias Analysis

**Figure 3:**
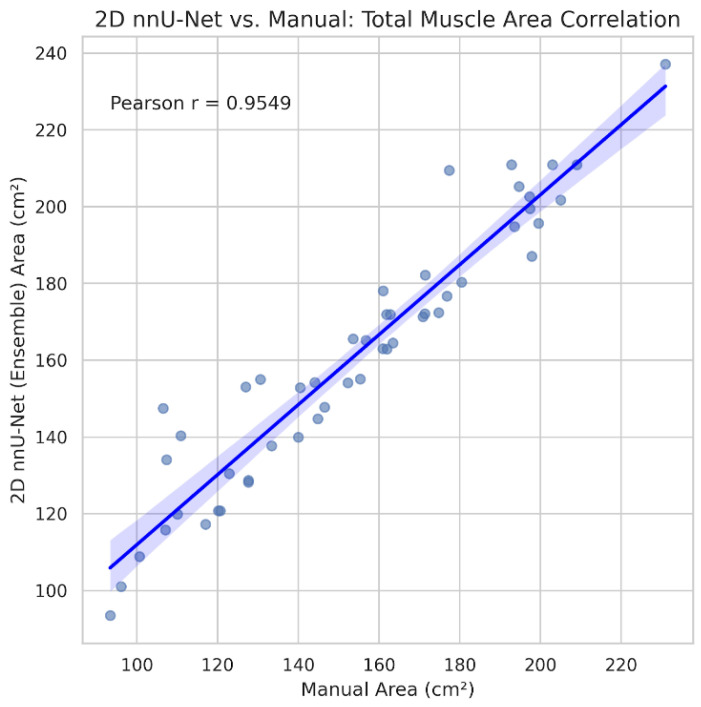
The scatter plot with linear regression (solid line) shows the correlation between total cross-sectional area (cm^2^) of the abdominal muscle, quantified by the 5-fold ensemble 2D nnU-Net, and the manual reference standard. The high Pearson correlation coefficient (r = 0.955) confirms the clinical interchangeability of the automated volumetric muscle assessment pipeline.

**Figure 4:**
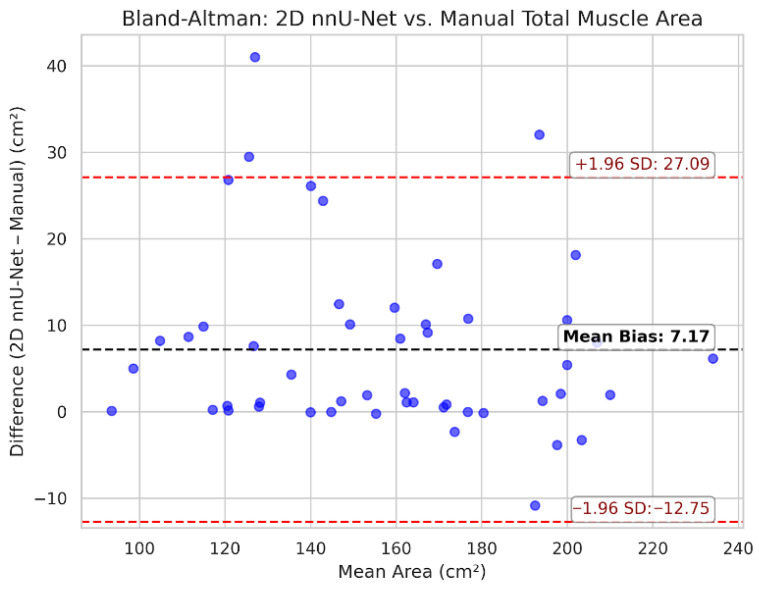
The Bland–Altman plot assesses the measurement bias between the automated pipeline (5-fold ensemble 2D nnU-Net) and manual segmentation. The black dashed horizontal line indicates the mean bias (+7.17 cm^2^), while dashed red lines represent the 95% limits of agreement. The marginal positive bias reflects the automated model’s systematic inclusion of thin fascial planes at the muscle-peritoneum interface.

Quantitative clinical metrics derived from the ensemble 2D nnU-Net predictions were compared against the manual reference standard. For total skeletal muscle cross-sectional area, Pearson correlation analysis demonstrated high agreement (r = 0.955, *p* < 0.001) with a mean absolute error (MAE) of 8.00 cm^2^ (Table 4) (Figure 3). Bland–Altman analysis for total muscle area revealed a mean bias of +7.17 cm^2^ (2D nnU-Net minus Manual), indicating a systematic, marginal increase in area quantification by the automated pipeline compared to the human rater (Figure 4).

**Figure 5:**
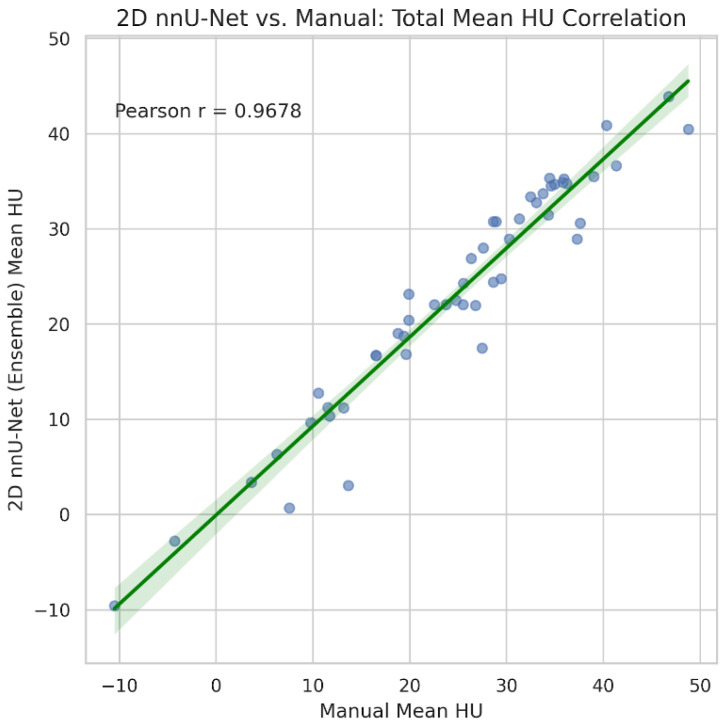
The scatter plot illustrates the high correlation (r = 0.968) between the 5-fold ensemble 2D nnU-Net and manual reference for mean muscle density (HU). Accurate automated HU extraction is critical for the downstream evaluation of myosteatosis.

Analysis of mean muscle attenuation (Hounsfield Units) also yielded high agreement between the automated model and the manual reference (r = 0.968), with an MAE of 2.33 HU and a minimal mean bias of −1.67 HU (Figure 5).

### 3.4. Intra-Observer Reliability and Time Efficiency

**Figure 6:**
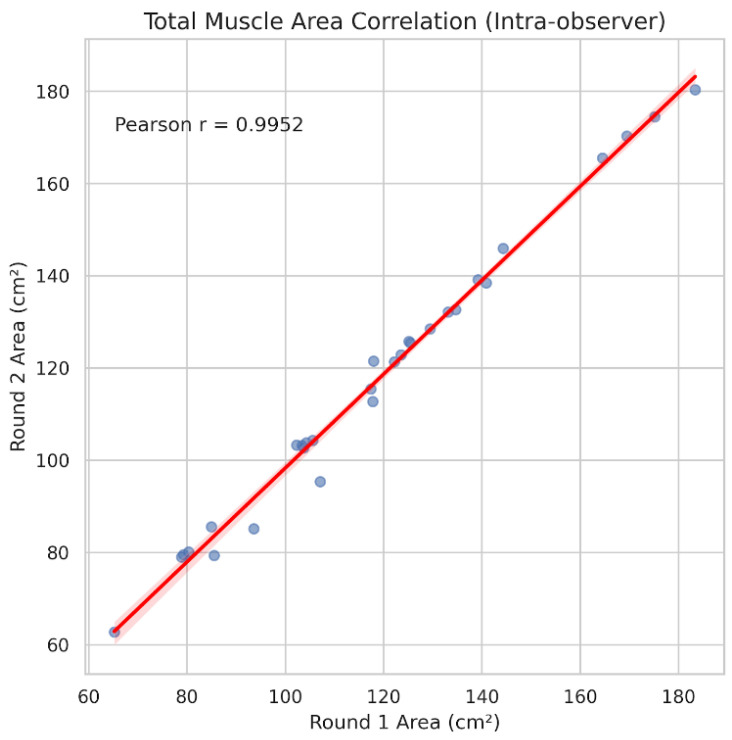
The scatter plot assesses the reproducibility of the manual segmentations (n = 30). The high Pearson correlation coefficient (r = 0.995) validates the stability of the reference standard utilized for external model evaluation.

**Figure 7:**
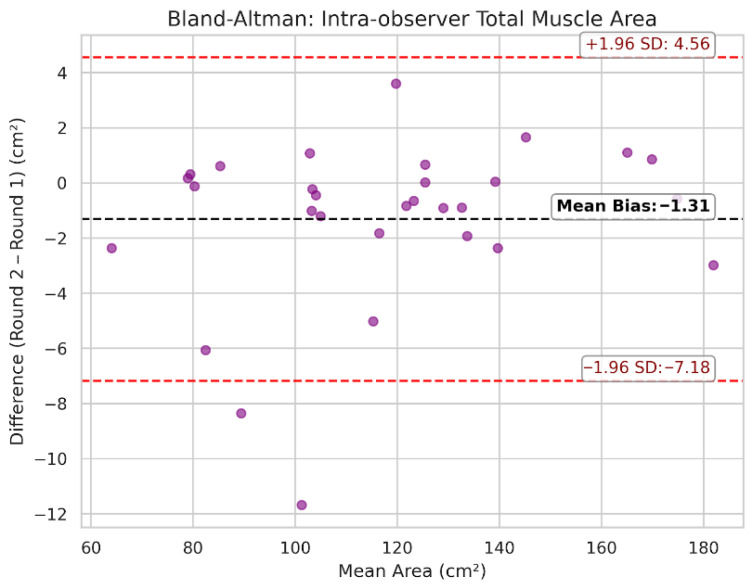
Bland–Altman plot for intra-observer total muscle area. The minimal mean bias (−1.31 cm^2^) and narrow limits of agreement indicate consistent performance by the human reader following a 14-day washout period, establishing a stringent performance ceiling.

The stability of the manual reference standard was confirmed via intra-observer analysis on a random subset of 30 cases. Agreement between the two independent rounds of manual segmentation, separated by the 14-day washout period, demonstrated a consistency for total muscle area (r = 0.995, *p* < 0.001) (Figure 6), with an MAE of 1.98 cm^2^ and a mean bias of −1.31 cm^2^ (Table 5) (Figure 7).

Regarding workflow efficiency, the manual segmentation process required 3 to 5 min of active human interaction per axial slice. In contrast, the automated 2D nnU-Net pipeline, executed via the custom Streamlit GUI, performed end-to-end inference, label map generation, and quantitative metric extraction in 3–5 s per case.

### 3.5. Inter-Observer Agreement

Inter-observer variability was evaluated in a subset of 30 cases from the initial AMOS22 training dataset, independently by a second reader using the same annotation workflow. Mean agreement across the three muscle classes was high, with an overall 3-class Dice coefficient of 0.909 ± 0.028. Class-specific Dice coefficients were 0.910 ± 0.034 for the psoas muscles, 0.926 ± 0.029 for the paraspinal muscles, and 0.890 ± 0.032 for the abdominal wall muscles. Quantitative agreement between readers was also strong for total skeletal muscle area (Pearson r = 0.988; MAE, 6.58 cm^2^), with similarly high agreement for class-specific area measurements (Table 6). These findings support the reproducibility of the manual reference framework and confirm that inter-reader variability is greater than intra-observer repeatability.

### 3.6. Qualitative Assessment and Error Analysis

**Figure 8:**
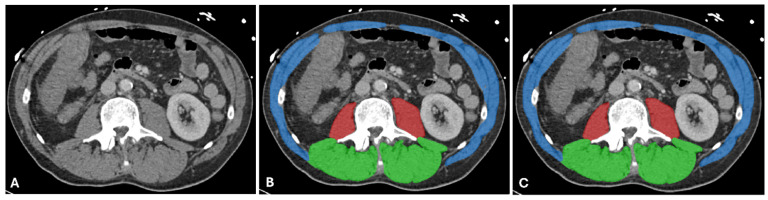
Upper-bound segmentation performance. Representative axial L3 CT slice from the external validation cohort demonstrating optimal model performance in standard cross-sectional anatomy. Panels display the unannotated axial CT (**A**), manual reference standard (**B**), and automated ensemble prediction (**C**). Target classes are color-coded (red = psoas, green = paraspinal, blue = abdominal wall). The overall mean DSC was 0.988. High spatial overlap was achieved across all individual classes: psoas (DSC: 0.992), paraspinal (DSC: 0.991), and abdominal wall (DSC: 0.980). The automated prediction closely aligns with the manual reference standard, without erroneously including adjacent tissues.

Visual inspection of the generated label maps aligned with the quantitative findings. In standard cross-sectional anatomies, the ensemble model demonstrated high anatomical fidelity, accurately delineating all three muscle compartments without encroaching on adjacent viscera (Figure 8). In the highest-performing external validation case, the ensemble model achieved a mean DSC of 0.988, confirming that near-complete spatial overlap was attainable when normal fascial planes and muscle boundaries were preserved.

**Figure 9:**
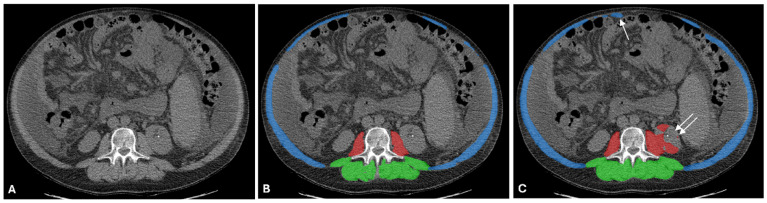
Lower-bound segmentation performance in complex anatomy. Representative axial L3 CT slice from the external validation cohort demonstrating limited model performance in a patient with ascites. Panels display the unannotated non-contrast CT (**A**), manual reference standard (**B**), and automated ensemble prediction (**C**). Target classes are color-coded (red = psoas, green = paraspinal, blue = abdominal wall). The overall mean DSC was 0.822. Psoas segmentation (DSC: 0.718) was limited by the inclusion of the isodense left kidney (double arrows) on unenhanced imaging. Abdominal wall segmentation (DSC: 0.817) included portions of the peritoneum adjacent to the atrophic rectus abdominis (single arrow). The paraspinal muscle group maintained a higher spatial overlap (DSC: 0.933).

**Figure 10:**
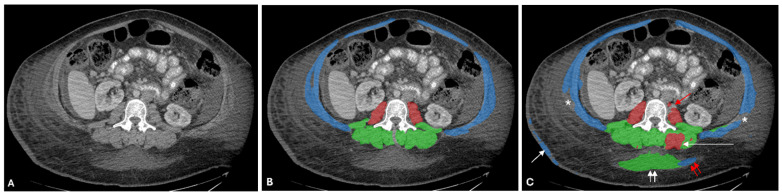
Algorithmic misclassification associated with subcutaneous edema. Representative axial L3 CT slice from the internal training dataset demonstrating localized boundary failures in a patient with altered soft tissue density. Panels display the unannotated CT (**A**), manual reference standard (**B**), and automated prediction (**C**). Target classes are color-coded (red = psoas, green = paraspinal, blue = abdominal wall). Spatial overlap was reduced across all classes, psoas (DSC: 0.655), paraspinal (DSC: 0.648), and abdominal wall (DSC: 0.744). The psoas prediction undersegments the left psoas (single red arrow) and bridges into the left paraspinal compartment (single long white arrow). The paraspinal boundary extends into the posterior subcutaneous edema (double short white arrows). The abdominal wall boundary undersegments the bilateral oblique muscles (*) and includes a focal region of left posterior edema (double short red arrows) and the right posterior skin (white arrow).

However, a qualitative review of subthreshold cases in the advanced liver disease cohort revealed specific patterns of anatomical ambiguity. In the lowest-performing external validation case, the mean DSC improved from 0.720 with the best single-fold model to 0.822 with the 5-fold ensemble, but the residual error pattern remained informative. In non-contrast scans of patients with severe ascites, localized boundary failures occurred due to the inclusion of isodense structures, such as the kidney or adjacent peritoneum (Figure 9). These lower-bound cases showed the greatest vulnerability in the psoas and abdominal wall compartments, whereas the paraspinal musculature remained relatively preserved. Furthermore, profound subcutaneous edema occasionally altered normal soft-tissue density gradients, leading to localized over-segmentation of edematous tissue or under-segmentation of the oblique musculature (Figure 10). Taken together, these findings indicate that model failure was not random, but clustered around reproducible anatomical scenarios in which fluid, edema, and severe muscle thinning obscured the true soft-tissue interface.

## 4. Discussion

This study demonstrates that a fully automated, ensemble-based 2D nnU-Net pipeline can achieve high quantitative accuracy and clinical reliability for L3 skeletal muscle segmentation. Validated against a highly stable, expert-derived reference standard (intra-observer r = 0.995), the ensemble model demonstrated high spatial overlap (mean DSC 0.937) and measurement agreement (r = 0.955 for total area) on a diverse, high-acuity external clinical cohort. Crucially, the implementation of a 5-fold ensemble inference strategy provided a measurable fail-safe in anatomically complex cases, significantly raising the minimum performance threshold compared to single-fold architectures. An additional inter-observer agreement using an independent reader further demonstrated reproducible manual segmentation performance, with a mean 3-class Dice coefficient of 0.909 and strong total-area agreement (r = 0.988). These data strengthen the reference-standard framework while also highlighting that reader-to-reader variability remains greater than same-reader repeatability. However, automated measurements should not be interpreted as strictly interchangeable with manual segmentation, as a Bland–Altman analysis showed a mean positive bias of 7.17 cm^2^ and an MAE of 8.00 cm^2^ for total skeletal muscle area. Accordingly, the present data support strong quantitative agreement rather than formal equivalence, and the effect of these discrepancies on derived indices such as skeletal muscle index warrants further clinical evaluation. Equally important, the principal advantage of the ensemble strategy was not an improvement in average DSC, but a reduction in lower-bound failure severity in the most anatomically challenging cases. From a clinical implementation perspective, this lower-tail stabilization may be more meaningful than marginal changes in mean performance, because rare but extreme segmentation failures are more likely to compromise safety and user trust.

A notable finding of this study is the high generalizability of the ensemble model, which was trained on a relatively constrained cohort of 164 cases and successfully validated on a highly complex external cohort. In deep learning, model robustness is traditionally associated with large-scale datasets comprising thousands of annotated studies. However, our results demonstrate that the quantity of raw data can be effectively offset by dataset heterogeneity and an optimized network architecture. The selected AMOS22 training pool exhibited broad pathological diversity and technical variance across four distinct scanner manufacturers. By employing extensive, dynamic data augmentation during training, the nnU-Net framework forces the network to learn fundamental anatomical features rather than overfitting scanner-specific artifacts [49]. In the present study, these augmentations were applied online within the standard nnU-Net v2 training pipeline rather than through manual offline dataset expansion, improving reproducibility and reducing dependency on investigator-specific augmentation design. Additional safeguards against overfitting included rotational five-fold cross-validation, dataset-specific CT normalization, and consensus-based ensemble inference across independently trained folds. Although these strategies cannot fully eliminate the risk of overfitting in a modestly sized dataset, the stable fold-wise performance, parallel training-validation convergence, and preserved external validation accuracy argue against substantial memorization of the training cases.

The efficacy of this training strategy was explicitly demonstrated in the external validation cohort, which comprised patients with advanced liver disease. A recent meta-analysis reported a pooled DSC of 0.941 for AI-based skeletal muscle segmentation across various studies [58]. While several contemporary deep learning models report higher spatial overlap metrics—such as DSCs of 0.97—these architectures are predominantly trained and validated on routine health check-up populations or general outpatients [64,65]. When automated pipelines are deployed on anatomically complex or diseased cohorts, performance predictably degrades. For instance, a previous U-Net model demonstrated a muscle DSC of 0.96 on a standard test set, which subsequently dropped to 0.92 when applied to a hepatocellular carcinoma cohort [39]. The external validation cohort in the present study, burdened by massive fluid shifts and profound soft-tissue edema, served as an intentional ‘stress test.’ By maintaining an overall mean DSC of 0.937 across unpredictable anatomical presentations (Figure 8), the 5-fold ensemble model demonstrates resilience against catastrophic boundary failures, proving its deployability in high-acuity clinical scenarios.

A critical objective of this study was to define the technical boundaries of the 2D nnU-Net. While the ensemble model achieved high average accuracy, threshold analysis (DSC ≥ 0.90) revealed distinct, class-specific anatomical vulnerabilities. The paraspinal musculature demonstrated high algorithmic resilience (98% of cases ≥ 0.90), likely because it is spatially anchored by rigid osseous landmarks (the vertebral body and transverse processes) that remain identifiable despite soft-tissue distortion. Conversely, the abdominal wall proved the most challenging target, falling below the 0.90 threshold in 32% of cases. This class-specific performance gradient is corroborated by previous body composition studies, which consistently identify the anterolateral abdominal wall as the most anatomically vulnerable compartment due to its natively thin muscular composition, highly variable fascial boundaries, and susceptibility to severe myosteatosis [45]. The psoas muscles showed intermediate performance, consistent with their relative anatomical compactness but vulnerability to boundary confusion with adjacent retroperitoneal structures, particularly on non-contrast studies with marked ascites.

Qualitative analysis of sub-optimal predictions (Figure 9) indicates that these relative failures are rarely arbitrary; rather, they are driven by severe, patient-specific pathological alterations. In patients with profound sarcopenia, the rectus abdominis and oblique muscles become extremely thin, causing the automated boundary to inadvertently capture adjacent peritoneal layers. Similarly, severe subcutaneous edema disrupts normal tissue gradients, prompting the algorithm to over-segment into the infiltrated adipose tissue (Figure 10). The psoas muscle exhibited localized vulnerabilities (16% of cases < 0.90) primarily on unenhanced imaging, particularly when combined with third-spacing fluid. As demonstrated in Figure 9, the presence of ascites on non-contrast CT renders the left psoas completely isodense with the adjacent left kidney, leading to a false-positive algorithmic bridging between the structures. These observations define the principal technical failure modes of the current model: (1) loss of contrast between the psoas and adjacent retroperitoneal structures on unenhanced scans, (2) erroneous inclusion of the peritoneal margin adjacent to markedly thinned abdominal wall musculature, and (3) over-segmentation into posterior subcutaneous edema when normal fascial boundaries are blurred.

Despite these complex localized failures, the sub-threshold cases for the abdominal wall and psoas still maintained mean DSCs of 0.836 and 0.857, respectively, proving that the ensemble architecture reliably prevents catastrophic, whole-image segmentation collapse even in severely distorted anatomies. Bland–Altman analysis revealed a systematic positive mean bias of 7.17 cm^2^ in total muscle area when comparing the ensemble model with the manual reference. Although this degree of bias remained small relative to the full range of observed muscle areas in the cohort, it is not negligible and should be interpreted cautiously when model-derived measurements are used for threshold-based categorization or longitudinal comparison. In the context of cross-sectional macroscopic anatomy, this marginal overestimation is a positive indicator of algorithmic consistency. The 2D nnU-Net reliably includes thin fascial planes and muscle-peritoneum interfaces that a human rater—acting with necessary caution to avoid inadvertently including visceral fat or bowel—might conservatively omit during manual tracing. This finding aligns with validation studies of fully automated body composition pipelines, which frequently report a high correlation combined with a slight positive volumetric bias, reflecting the AI’s persistent inclusion of intermuscular tissue that human readers tend to under-segment [48]. Nevertheless, the existence of lower-bound cases with reduced Dice performance underscores that qualitative review and human oversight remain important when the model is applied to anatomically extreme or pathologically distorted examinations.

The transition from conventional semi-automated thresholding to this fully automated DL pipeline addresses the primary bottleneck in body composition research. Threshold-based tools require extensive manual correction when HU values are distorted by myosteatosis or adjacent pathology. By providing a semantic understanding of the anatomy, the 2D nnU-Net distinguishes between adjacent structures regardless of HU overlap. Integrated into a custom Streamlit GUI, this pipeline reduced the time required for complete segmentation, metric extraction, and reporting from 3 to 5 min to merely 3 to 5 s per case. This processing speed strictly aligns with the performance of recently implemented fully automated AI screening systems, which report mean processing times of approximately 4 s from CT acquisition to final report generation [65]. Accordingly, the present study supports the use of this pipeline as a rapid quantitative measurement tool for L3 skeletal muscle analysis. However, we did not evaluate downstream sarcopenia classification thresholds, diagnostic accuracy, or associations with clinical outcomes, and these applications should be considered future validation targets rather than established findings of the current work. Similarly, although the current results support technical feasibility and workflow efficiency, prospective testing of workflow integration, user adoption, and real-world clinical benefit was not conducted and should not be inferred from the present retrospective analysis. Within this framework, the most immediate clinical role of the current tool would be to provide rapid, standardized L3 muscle measurements that could support sarcopenia screening, longitudinal monitoring of body composition, and future risk-stratification models in high-risk patient populations. Whether such automated measurements ultimately influence treatment selection, procedural planning, morbidity, or mortality will require dedicated prospective outcome-based validation.

This study has several limitations. First, despite a multi-institutional training source, the sample size—164 training slices and 50 external validation cases—is modest relative to population-scale DL studies with thousands of annotated exams [64]. Although augmentation and an ensemble strategy improved performance, generalizability should be confirmed in larger, multicenter, high-acuity cohorts. The relatively limited training size also introduces residual overfitting risk, even though five-fold cross-validation, dynamic augmentation, nnU-Net-based normalization, and ensemble inference were used to reduce model dependence on any single partition. Accordingly, the present findings should be interpreted as evidence of technical feasibility and external proof of concept rather than definitive population-level generalizability. Second, the external validation cohort was intentionally enriched for advanced liver disease to stress-test performance under severe anatomical distortion (e.g., ascites, edema), introducing spectrum bias and limiting applicability to routine opportunistic screening. Although the training cohort incorporated multi-vendor and multi-institutional heterogeneity, robustness was not formally evaluated using predefined subgroup analyses stratified by scanner vendor, acquisition protocol, reconstruction parameters, or contrast phase. Because the external validation cohort was derived from a single institution, the present results should not be interpreted as a dedicated protocol-specific or vendor-specific robustness assessment. Future validation should include broader clinical indications, disease severities, and body habitus across institutions. Third, the pipeline operates on a single 2D axial L3 slice. While L3 is a validated surrogate for whole-body muscle mass, single-slice analysis is an approximation and sensitive to inter-individual anatomic variability; 3D volumetric and/or multi-slice approaches merit evaluation [48]. Although incomplete L3 slices were excluded before annotation and analysis, the present study did not include a formal image-quality grading system for motion, noise, or beam-hardening artifact severity. Fourth, reported processing times for manual versus automated workflows are observational estimates rather than outputs of a formal time–motion study; prospective measurement should quantify mean time savings, variability, and cost/throughput effects in high-volume settings. Finally, the retrospective, single-institution design limits inferences about effects on clinical decision-making and patient outcomes. The present analysis also did not assess downstream classification accuracy for sarcopenia, prognostic stratification, or outcome prediction based on model-derived measurements. Accordingly, although the current results support technical validity and workflow feasibility, they should not be interpreted as evidence of prospective clinical benefit or implementation readiness without further real-world validation. Prospective deployment studies are needed to assess real-time impact and patient-centered outcomes.

## 5. Conclusions

The developed ensemble-based 2D nnU-Net pipeline provides an accurate, reliable, and time-efficient solution for L3 skeletal muscle segmentation. By training on a heterogeneous dataset and employing a 5-fold ensemble strategy, the model effectively mitigates catastrophic segmentation failures in anatomically complex patients. Integrated with a custom GUI, this automated tool eliminates the labor-intensive bottlenecks of manual segmentation, enabling scalable quantitative assessment of L3 skeletal muscle and related body composition measurements in routine clinical practice. Its role as a downstream tool for sarcopenia classification or outcome prediction remains to be established in future prospective studies.

## Figures and Tables

**Table 1:** Demographics and clinical characteristics of the external validation cohort.

Characteristic	Value
Total Cases (n)	50
Age (years)	63 ± 15
Sex (Male:Female)	18:32
Primary Clinical Indications (n, %)	
- Liver cirrhosis	11 (22%)
- Routine evaluation for TIPS	10 (20%)
- Refractory ascites	9 (18%)
- Hepatocellular carcinoma	8 (16%)
- Portosystemic shunt	6 (12%)
- PV thrombosis evaluation	5 (10%)
- Hepatic hydrothorax	3 (6%)
- Axial CT Findings at L3	
- Ascites	44 (88%)
- Subcutaneous edema	3 (6%)
- Liver masses	1 (2%)
- No ascites	6 (12%)

**Table 2:** Internal 5-Fold Cross-Validation Segmentation Performance.

Fold	Psoas	Paraspinal	Abdominal Wall	Mean DSC
0	0.932	0.940	0.898	0.924
1	0.939	0.956	0.923	0.939
2	0.941	0.952	0.908	0.934
3	0.925	0.935	0.894	0.918
4	0.946	0.958	0.919	0.941
Mean	0.937	0.948	0.908	0.931 ± 0.010

DSC = Dice similarity coefficient.

**Table 3:** External validation: single-fold vs. 5-fold ensemble architecture.

Metric	Single Fold (Fold 4)	5-Fold Ensemble	*p*-Value
Overall mean DSC	0.937	0.937	0.736
Psoas DSC	0.939	0.941	0.448
Paraspinal DSC	0.960	0.960	0.810
Abdominal wall DSC	0.912	0.911	0.858
Minimum observed DSC	0.720	0.822	—

**Table 4:** Clinical measurement agreement: 2D nnU-Net vs. manual reference.

Metric	Pearson r	MAE	Mean Bias (nnU-Net—Manual)
Total muscle area (cm^2^)	0.955	8.00	+7.17
Total mean attenuation (HU)	0.968	2.33	−1.67

**Table 5:** Intra-observer reliability for the manual reference standard.

Metric	Pearson r	MAE	Mean Bias (Round 2–Round 1)
Total muscle area (cm^2^)	0.995	1.98	−1.31
Total mean attenuation (HU)	0.995	0.74	+0.56

**Table 6:** Inter-observer agreement for the manual reference standard.

Metric	Pearson r	MAE	Mean Bias (Reader 2–Reader 1)
Total muscle area (cm^2^)	0.988	6.58	+5.71
Total mean attenuation (HU)	0.989	2.25	+1.90

## Data Availability

The AMOS22 multi-institutional dataset utilized for model training is publicly accessible at https://amos22.grand-challenge.org/. The custom Streamlit application template for image annotation, end-to-end model inference, and quantitative clinical reporting is open source and publicly available on GitHub at https://github.com/hyeonyu-IR/segmentation-label-app, accessed on 6 May 2026 (version 1.0).
